# Isolation of *Escherichia coli* O157:H7 from Intact Colon Fecal Samples of Swine^1^

**DOI:** 10.3201/eid0903.020350

**Published:** 2003-03

**Authors:** Ingrid Feder, F. Morgan Wallace, Jeffrey T. Gray, Pina Fratamico, Paula J. Fedorka-Cray, Rachel A. Pearce, Jeffrey E. Call, Richard Perrine, John B. Luchansky

**Affiliations:** * U.S. Department of Agriculture, Wyndmoor, Pennsylvania, USA; † U.S. Department of Agriculture, Athens, Georgia, USA; ‡ National Food Centre, Dublin, Ireland

**Keywords:** *Escherichia coli* O157, H7, feces, swine, dispatch

## Abstract

*Escherichia coli* O157:H7 was recovered from colon fecal samples of pigs. Polymerase chain reaction confirmed two genotypes: isolates harboring the *eaeA*, *stx*__1__, and *stx*__2__ genes and isolates harboring the *eaeA*, *stx*__1__, and *hly*_933_ genes. We demonstrate that swine in the United States can harbor potentially pathogenic *E. coli* O157:H7.

During the past two decades, disease caused by *Escherichia coli* O157:H7 has been increasing (*1*). Currently, the Centers for Disease Control and Prevention estimates that *E. coli* O157:H7 causes an average of 500 outbreaks that affect >73,000 persons and result in >61 deaths each year in the United States (*2*). The epidemiology of *E. coli* O157:H7 has become an important research topic as manure harboring *E. coli* O157:H7 is dispersed, and soil, food, and water are cross-contaminated with feces containing *E. coli* O157:H7 (*1**,**3*). Although cattle feces are the most important source of *E. coli* O157:H7, the need to evaluate the presence of *E. coli* O157:H7 in the feces of other animal species has been recognized (*1*). The presence of *E. coli* O157:H7 in swine feces has been reported in Japan (*4*), Norway (*5*), and Chile (*6*); however, to date, *E. coli* O157:H7 has not been reported in swine in the United States.

## The Study

Colon samples were collected at a cooperating swine slaughter facility from 305 swine carcasses during evisceration. Two to three inches of distal colon that contained feces at the first point proximal to the rectum was resected and maintained on ice for approximately 2 hours before processing (Figure). Ten grams of feces from each colon was transferred to filter-lined sterile plastic bags. One hundred milliliters of brilliant green bile broth (Difco Laboratories, Detroit, MI), prewarmed to 37°C, was added to each filter stomacher bag containing feces and incubated at 37°C for 6 h with shaking (150 rpm) (*7*). After enrichment, 1.0-mL aliquots were processed by using Dynabeads anti–*E. coli* O157 (Dynal Biotech, Oslo, Norway), according to manufacturers’ instructions with modification. Bead/sample suspensions were incubated at room temperature for 30 min with continuous mixing on a Bellco roller drum (Bellco Glass, Inc., Vineland, NJ) before plating onto sorbitol MacConkey (SMAC; Difco Laboratories), cefixime/tellurite (CT; cefixime-tellurite supplement, Dynal Biotech)‑SMAC agars, and rainbow agar O157 (Biolog, Inc., Hayward, CA). Black colonies from rainbow agar O157 and sorbitol-negative colonies from CT-SMAC and SMAC agars were tested for the absence of ß-glucuronidase and the ability to ferment lactose by using *E. coli* broth containing 4-methlumbelliferyl-β-D-glucuronide (MUG) (EC medium with MUG; Difco Laboratories) and MacConkey broth (Difco Laboratories), respectively. Lactose-positive/MUG-negative isolates were serotyped by using the *RIM E. coli* O157:H7 Latex Test (Remel, Lenexa, KS). Up to 10 *E. coli* O157 latex agglutination–positive isolates per colon fecal sample were tested for the presence of the *rfb*_O157_ gene by using polymerase chain reaction (PCR) (*8*). Isolates positive for the *rfb*_O157_ gene were further characterized for the presence of genes encoding for the H7 flagellar protein (*fliC*_H7_), Shiga toxin 1 (*stx_1_*), Shiga toxin 2 (*stx_2_*), intimin protein (*eaeA)*, and hemolysin (*hly*_933_) (*9*). We conducted further analysis using antimicrobial resistance patterns, pulsed-field gel electrophoresis (PFGE), and ribotyping on all *E. coli* O157 PCR–positive isolates containing *fliC*_H7_, *stx_1_*, *stx_2_*, *eaeA*, or *hly*_933_. However, for tabulation purposes, each sample ultimately contributed one isolate. When *fliC*_H7_, *stx_1_*, *stx_2_*, *eaeA*, or *hly*_933_ was not detected in PCR-confirmed *E. coli* O157 isolates, further analysis was performed on only one *E. coli* O157 isolate per colon sample.

**Figure:**
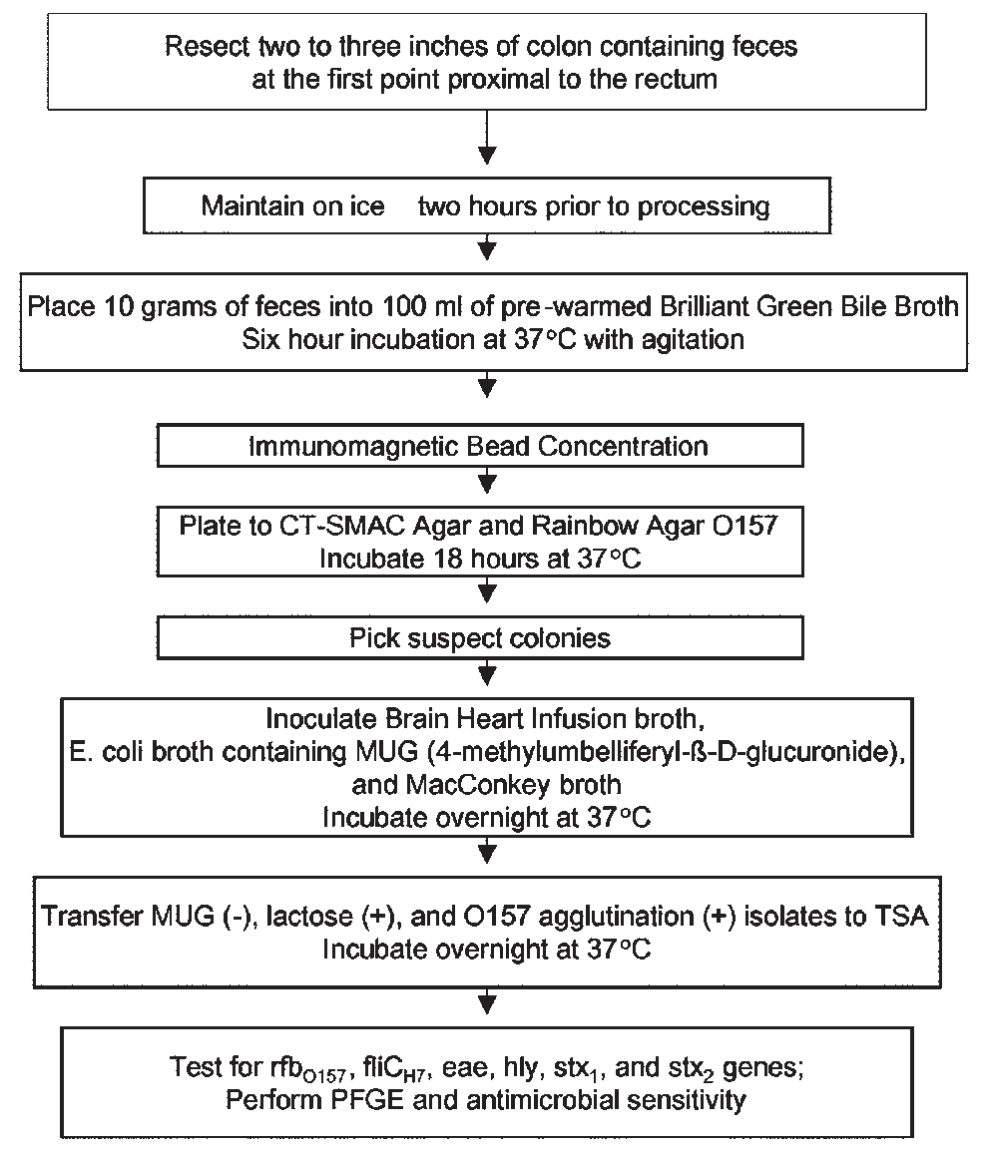
Procedure for isolating *Escherichia coli* O157 from swine colon fecal samples.

*E. coli* O157 isolates were tested for susceptibility to 17 antimicrobial agents (amikacin, amoxicillin/clavulanic acid, ampicillin, apramycin, cefoxitin, ceftriaxone, cephalothin, chloramphenicol, ciprofoxacin, gentamicin, imipenem, kanamycin, nalidixic acid, streptomycin, sulfamethoxazole, tetracycline, and trimethoprim/sulfamethoxazole) as described (*10*) by using a custom-made semiautomated broth microdilution assay (Sensititre, Trek Diagnostics, Westlake, OH). Imipenem was used at concentrations of 0.25–8.0 μg with the following breakpoints: sensitive (<4) and resistant (≥16).

For PFGE, DNA was digested with 50 U *Xba*I (Invitrogen Corp., Carlsbad, CA) for 4 h at 37°C. PFGE was performed by using a CHEF Mapper XA system (Bio-Rad, Hercules, CA) at 14°C with pulses ramping from 2.16 s to 63.8 s over 18 h. PFGE patterns were evaluated visually, and isolates were assigned to the same pulsotype when exhibiting a difference of <3 bands from the index isolate. Ribotyping of the *E. coli* O157 isolates was done by using a RiboPrinter (Qualicon, Inc., Wilmington, DE) as described in the user’s manual. Restriction digests were performed on *E. coli* O157 isolates by using the *Eco*RI enzyme (Qualicon, Inc.).

A total of 305 colon samples were randomly collected on 8 different days over a 6-month period as follows: collection day 1 (February 16, 2001), n=5; collection day 2 (March 8, 2001), n=20; collection day 3 (March 22, 2001), n=40; collection day 4 (April 20, 2001), n=40; collection day 5 (May 4, 2001), n=50; collection day 6 (May 16, 2001), n=50; collection day 7 (June 20, 2001), n=50; and collection day 8 (July 10, 2001), n=50 (Table). Eighteen (5.9%) of the 305 colon samples had isolates positive for *rfb*_O157_. Isolates from 6 of these 18 colon samples also contained *fli*C_H7_. Two gene combinations based on the presence or absence of *stx*_1_, *stx*_2_, *eae*, and *hly*_933_ were detected in these *E. coli* O157:H7 PCR-confirmed isolates. The *stx*_1_, *eaeA*, and *hly*_933_ virulence pattern was detected in two isolates (isolates1 and 2) from two of the five colon samples collected on February 16, 2001, and the *stx*_1_, *stx*_2_, and *eaeA* virulence pattern was detected in 22 isolates (isolates 6–27) from 4 of the 50 colon samples collected on May 4, 2001. None of the *E. coli* O157:H7 isolates recovered contained all four of the virulence genes (*stx*_1_, *stx*_2_, *eaeA*, and *hly*_933_). None of the *E. coli* O157:non-H7 isolates (isolates 3–5, 28–36) in the present study contained *stx*_1_, *stx*_2_, *eae*, or *hly* genes. Non–Shiga toxin–producing *E. coli* O157:non-H7 isolates have been previously isolated from the feces of pigs (*11*,*12*). For slaughterhouse visits on March 8, March 22, June 20, and July 10, 2001, *E. coli* O157 and *E. coli* O157:H7 were not recovered from any of the colons sampled.

**Table:** Characterization of *Escherichia coli* O157:H7 and non-H7 isolates recovered from 305 swine fecal colon samples^a,b^

Collection date	Swine *E. coli* O157 isolate no.	*E. coli* O157 positive colon samples/total colon samples collected	Colon ref. no.	No. of isolates recovered from sample	PCR characteristics	*E*. *coli* O157 ribotyping	PFGE type	Antimicrobial resistance pattern^b^	
Feb. 16, 2001	1	2/5	1	1	*rfb*_O157_, *fli*C_H7_, *stx*_1_, *eae*, *hly*_933_	H7	1	Pan-sensitive	
	2		2	1	*rfb*_O157_, *fli*C_H7_, *stx*_1_, *eae*, *hly*_933_	H7	1	Pan-sensitive	
								
Mar. 8, 2001	No isolates	0/20							
								
Mar. 22, 2001	No isolates	0/40							
								
Apr. 20, 2001	3	3/40	3	1	*rfb*_O157_	Non-H7	3	kan, strept, sulfa, tet	
	4		4	1	*rfb*_O157_	Non-H7	3	kan, strept, sulfa, tet	
	5		5	1	*rfb*_O157_	Non-H7	3	kan, sulfa, tet	
								
May 4, 2001	6–11	4/50	6	6	*rfb*_O157_, *fli*C_H7_, *stx*_1_, *stx*_2_, *eae*	H7	2	Pan-sensitive	
	12–16		7	5	*rfb*_O157_, *fli*C_H7_, *stx*_1_, *stx*_2_, *eae*	H7	2	Pan-sensitive, except isolate no. 15 resistant to strept	
	17–25		8	9	*rfb*_O157_, *fli*C_H7_, *stx*_1_, *stx*_2_, *eae*	H7	2	Pan-sensitive	
	26,27		9	2	*rfb*_O157_, *fli*C_H7_, *stx*_1_, *stx*_2_, *eae*	H7	2	Pan-sensitive	
								
May 16, 2001	28	9/50	10	1	*rfb*_O157_	Non-H7	4	Pan-sensitive	
	29		11	1	*rfb*_O157_	Non- H7	4	tet	
	30		12	1	*rfb*_O157_	Non-H7	4	tet	
	31		13	1	*rfb*_O157_	Non-H7	4	strept, tet	
	32		14	1	*rfb*_O157_	Non- H7	4	tet	
	33		15	1	*rfb*_O157_	Non-H7	4	tet	
	34		16	1	*rfb*_O157_	Non- H7	4	tet	
	35		17	1	*rfb*_O157_	Non- H7	4	tet	
	36		18	1	*rfb*_O157_	Non-H7	4	tet	
								
June 20, 2001	No isolates	0/50							
								
July 10, 2001	No isolates	0/50							
								
	Total=36	18/305	18						

^a^ Each isolate listed in the following table represents an isolate from an individual colon sample. ^b^Feb., February; Mar., March; Apr., April; ref., reference; PCR, polymerase chain reaction; PFGE, pulsed-field gel electrophoresis; kan, kanamycin; strept, streptomycin; sulfa, sulfamethoxazole; tet, tetracycline.

All *E. coli* O157:H7 isolates recovered in this study were sensitive to the antimicrobial agents tested, with the exception of one isolate (isolate 15) that was resistant to streptomycin. This isolate was recovered from a colon from which a pan-sensitive *E. coli* O157:H7 was also recovered. The antimicrobial sensitivity pattern of the *E. coli* O157:non-H7 isolates was more varied than that of the *E. coli* O157:H7 isolates with five different susceptibility patterns noted. Only one of the *E. coli* O157:non-H7 isolates was pan-sensitive. These data are similar to previous reports in which antimicrobial resistance among *E. coli* O157 non–Shiga toxin–producing isolates was higher than that of Shiga toxin–producing *E. coli* O157 isolates (*11*).

As previously shown, ribotyping did not discriminate among isolates within the *E. coli* O157:H7 serotype (*13*). Additionally, the *E. coli* O157:non-H7 isolates were indistinguishable from one another. Four PFGE profiles were noted. The *E. coli* O157:H7 isolates obtained from colon 1 and colon 2 on February 16, 2001, exhibited the PFGE type 1 pattern, whereas the *E. coli* O157:H7 isolates obtained from four colons on May 4, 2001 exhibited the PFGE type 2 pattern. The *E. coli* O157:non-H7 isolates obtained on April 20, 2001, and May 16, 2001, exhibited PFGE patterns 3 and 4, respectively.

## Conclusion

Results from this study demonstrate that pigs in the United States can harbor *E. coli* O157:H7. The recovery rate of *E. coli* O157:H7 from colon fecal samples of pigs reported in this study was 2.0% (6/305). Previous attempts to isolate *E. coli* O157:H7 from swine feces in the United States have been unsuccessful (*12*,*14*). Use of more appropriate methods for sampling, processing, and culturing swine feces may have accounted for the ability to recover and isolate *E. coli* O157:H7 from swine feces in our study. For example, samples were obtained from the colon, transported on ice, and processed within 2 h of collection. The absence of antibiotics in our enrichment step may have also facilitated the recovery of *E. coli* O157:H7 from swine feces. Furthermore, although direct comparisons cannot be made between cattle studies, the recovery rate of Shiga toxin–producing *E. coli* O157 from cattle feces has improved over the past 10 years. This is most likely due to more conducive sampling procedures, culture practices, and detection methods than an increase in true carriers. The detection of *E. coli* O157 in swine feces has previously been based on the isolation techniques used for the recovery of *E. coli* O157 from cattle feces. The difficulty in detecting *E. coli* O157 from swine feces may in part be attributable to differences in the physiologic environment between swine and cattle feces. More appropriate isolation techniques may still be discovered for detecting *E. coli* O157 in swine.

Although our recovery rates of *E. coli* O157:H7 from swine are similar to recovery rates in Japan (*4*), we recovered a genotype in addition to the *stx*_1_, *stx*_1_, and *eaeA* genotype: the *stx*_1_, *eaeA*, and *hly*_933_ genotype. In Norway, the recovery rate (0.1%) of *E. coli* O157:H7 from pig feces was much lower (*5*). Isolates recovered from Norway possessed the *stx*_2_ and *eae* genes only; however, the presence of the *hly* gene was not determined (*5*).

The ability to produce one or more Shiga toxins is an important virulence characteristic of *E. coli* O157:H7 (*1*). However, production of Shiga toxins alone may not be sufficient for *E. coli* O157:H7 to be pathogenic (*1*). Other virulence factors such as the intimin protein (involved in the attachment of the *E. coli* O157 to enterocytes), the presence of a plasmid-encoded hemolysin, or both, are important in the pathophysiology of hemorrhagic disease (*1*). *E. coli* O157:H7 isolates recovered in this study possessed either two virulence factors, *eaeA* and *hly*_933_, in addition to *stx*_1_ or one virulence factor, *eaeA*, in addition to *stx*_1_ and *stx*_2_. These isolates can potentially cause disease and should be considered pathogenic to humans. Since human *E. coli* O157:H7 clinical isolates contain the *stx*_1_, *stx*_2_, *eae*A, and *hly* genes, the human pathogenicity of *E. coli* O157:H7 isolates from pigs that lack the *hly* gene requires further study.

The clonal nature of the isolates that were isolated on a particular day suggests transmission of *E. coli* O157 between pigs. Unfortunately, we did not have access to information concerning the source of the pigs from which the samples were collected, the number of pigs slaughtered from a given farm, or the holding facilities and grouping of the pigs before slaughter. Therefore, we do not know whether *E. coli* O157 transmission between pigs occurred on the farm, in transit, or while the pigs were in a holding pen at the slaughterhouse.

This study did not permit inferences of *E. coli* O157:H7 isolation rates with respect to the season, nor can inferences of *E. coli* O157:H7 isolation rates be made with respect to swine or herd prevalence. The relatively low recovery rate of *E. coli* O157:H7 from swine feces compared to cattle feces warrants further study to determine the significance and prevalence of *E. coli* O157:H7 in swine and if different enrichment and isolation methods would have an impact on the recovery of *E. coli* O157:H7 from swine feces. In addition, future studies should be conducted to determine the occurrence of *E. coli* O157 on swine hides, in swine mouths, and in swine stomachs.
